# Inhibition of NOX4/ROS Suppresses Neuronal and Blood-Brain Barrier Injury by Attenuating Oxidative Stress After Intracerebral Hemorrhage

**DOI:** 10.3389/fncel.2020.578060

**Published:** 2020-11-13

**Authors:** Jiayu Xie, Enhui Hong, Baiyun Ding, Weiping Jiang, Shizhong Zheng, Zhichong Xie, Dan Tian, Yizhao Chen

**Affiliations:** ^1^Guangdong Provincial Key Laboratory on Brain Function Repair and Regeneration, Department of Neurosurgery, Zhujiang Hospital, The National Key Clinical Specialty, The Neurosurgery Institute of Guangdong Province, The Engineering Technology Research Center of Education Ministry of China, Southern Medical University, Guangzhou, China; ^2^Department of Neurosurgery, The First Affiliated Hospital of University of South China, Hengyang, China; ^3^Department of Neurosurgery, The Seventh Affiliated Hospital, Sun Yat-sen University, Shenzhen, China; ^4^Department of Neurosurgery, Jinshazhou Hospital of Guangzhou University of Chinese Medicine, Guangzhou, China; ^5^Department of Hematology, Zhujiang Hospital, Southern Medical University, Guangzhou, China; ^6^Department of Neurosurgery, The First Affiliated Hospital of Guangzhou Medical University, Guangzhou Medical University, Guangzhou, China

**Keywords:** ICH = intracerebral hemorrhage, oxidative stress, blood-brain barrier, apoptosis, NOX4 NADPH oxidase

## Abstract

Intracerebral hemorrhage (ICH) is a common and severe neurological disorder that can effectively induce oxidative stress responses. NADPH oxidase 4 (NOX4) is a member of the NOX family of oxidases. It is expressed in the brain normally and involved in cell signal transduction and the removal of harmful substances. In some pathological conditions, it mediates inflammation and the aging of cells. However, few studies have focused on whether NOX4 is involved in brain injury caused by ICH. Therefore, this study aimed to clarify the role of NOX4 in the pathological process that occurs after ICH and the potential mechanism underlying its role. A rat model of ICH was established by the injection of collagenase type IV, and the expression of NOX4 was then determined. Further, siRNA-mediated protein expression knockdown technology was used for NOX4 knockdown, and western immunoblotting, immunohistochemistry, immunofluorescence, enzyme-linked immunosorbent assay (ELISA), and other molecular biological techniques were performed to assess the effects of NOX4 knockdown. Neurobiological scoring, brain water content determination, and other brain injury detection methods were also performed to assess the role of NOX4 following ICH. We found that the expression of NOX4 increased in the brains of rats after ICH, and that it was mainly expressed in neurons, astrocytes, vascular endothelial cells and microglia. Following NOX4 knockdown, the level of oxidative stress in the brain decreased considerably, the neurobehavioral scores improved, the levels of neuronal apoptosis reduced markedly, and the impairment of blood-brain barrier function was significantly ameliorated in rats with ICH. In conclusion, this study suggests that NOX4 expression is upregulated after ICH, which may cause an imbalance in the oxidative stress of relevant cells in the brain, leading to subsequent apoptosis of neurons and damage to the blood-brain barrier due to secondary brain injury following ICH.

## Introduction

Intracerebral hemorrhage (ICH) is a serious cerebrovascular disease with a high rate of mortality, and is the most severe subtype of stroke, accounting for about 15–20% of all stroke cases (Ribo and Grotta, 2006). Although increasing attention has been paid to research on ICH in recent years, to date, no effective targeted therapy has been established. Haematomas not only directly damage peripheral brain tissues, but also cause secondary brain injury (SBI). SBI includes an enhanced inflammatory response, increased levels of oxidative stress, persistent oedema caused by destruction of the blood-brain barrier, and apoptosis caused by various adverse factors (Wang et al., 2018). Accumulating evidence suggests that SBI is closely associated with the deterioration of neural functions after ICH and correlates with prognosis (Murthy et al., 2015). Therefore, the focus of our study was the alleviation of SBI following ICH.

Oxidative stress is considered an important factor mediating SBI (Ma et al., 2018b). In particular, reactive oxygen species (ROS) are molecules that lead to increased levels of oxidative stress and include superoxide anions (${\text{O}}_{2}^{-}$), hydroxyl radicals (OH^−^), and hydrogen peroxide (H_2_O_2_). Importantly, they can significantly affect the severity and progression of brain injury (Rani et al., 2016). Although ROS are physiologically maintained at low concentrations during cell transcriptional regulation, growth, and signal transduction, they can be rapidly transformed into harmful biological oxidants under pathological conditions (Mona et al., 2013).

As one of the main sources of ROS in mammals, the NADPH oxidase/NOX enzyme system is the only known system whose main function is to produce ROS (Brown and Borutaite, 2012; Haslund-Vinding et al., 2017). Therefore, targeted inhibition of the NADPH oxidase/NOX family may reverse the level of oxidative stress in brain tissues after ICH, thereby limiting the severity of SBI following ICH. To date, there are seven known NOX subtypes: NOX1-5 and DUOX1/2. These vary with regard to their distribution, structure, and function. Among them, NOX4 is one of the most extensively distributed isoforms in the central nervous system, which activates other cytoplasmic regulatory molecules (Radermacher et al., 2013; Craige et al., 2015). In addition, the downstream expression product of NOX4 is H_2_O_2_. As a ROS, it is more stable and more diffusive than ${\text{O}}_{2}^{-}$ and OH^−^ in organisms. NOX4 is also the most abundantly expressed NOX subunit, and its activity is the highest in vascular endothelial cells (Brown and Borutaite, 2012; Gray and Jandeleit-Dahm, 2015; Vendrov et al., 2015). Although NOX4 is indispensable in ROS signal transduction, its abnormal expression or overactivation may lead to tissue damage. Previous studies have shown that the expression of NOX4 in ischemic stroke and traumatic brain injury is closely related to the severity of SBI (Kleinschnitz et al., 2010; Casas et al., 2017; Ma et al., 2018a). However, the role of NOX4 in ICH is yet to be explored.

In this study, we examined the abnormal expression of NOX4 in brain tissues post ICH, and then determined its cellular distribution. *NOX4* siRNA was used to confirm the effects of NOX4 on ICH-induced oxidative stress. Subsequent functional and behavioral analyses, such as neurological and blood-brain barrier functional tests, as a prognostic indicator of ICH, were also measured. We investigated the role of NOX4 on oxidative stress, neuronal apoptosis, and blood-brain barrier injury following SBI after ICH, paving the road for a theoretical basis for the occurrence and development of SBI. Our findings may promote further investigations on the expression patterns and functions of NOX4 after ICH, enabling the development of novel approaches for the treatment of ICH.

## Materials and Methods

### Animals and Ethical Approval

This study included nearly 400 adult male Sprague-Dawley (SD) rats weighing 280–320 g. The SD rats were purchased from the Laboratory Animal Center of Southern Medical University (Guangzhou, Guangdong, China). All experiments were conducted in accordance with the National Institute of Health's guide for the care and use of laboratory animals. The animal experiments were approved by the ethics committee of Southern Medical University. Rats were housed under a standard 12-h light-dark cycle under specific-pathogen-free conditions, and were allowed free access to food and water. All rats used in this study were healthy and immune-normal. A power analysis was performed in order to reduce the number of animals used in these experiments.

### ICH Model

Rats were anesthetized with a pentobarbital intraperitoneal injection (45 mg/kg; stock: 0.3 g sodium pentobarbital dissolved in 100 ml of sterile normal saline), and collagenase type IV (0.2 U in 1 μl of sterile normal saline) was injected into the right basal ganglia, as previously reported (Schlunk et al., 2015). The stereoscopic coordinates were 3.5 mm lateral and 1.5 mm anterior to the anterior fontanelle, at a depth of 6.0 mm from the bone surface. The collagenase was injected into the right basal ganglia slowly within 10 min using a microsyringe (Shanghai high pigeon industry & trade co., LTD, Shanghai, China). The needle was held in place for 10 min after the injection to prevent back-flow (Zeng et al., 2017). The burr holes in the skull were sealed with bone wax and the wound was stitched. In the Sham group, 0.4 μl of normal saline was injected into the right basal ganglia using the same approach as that described for the treatment group. Anesthesia was maintained, and the rats were monitored throughout the procedure, including when the needle was held in place for 10 min. The rats were allowed to recover in squirrel cages with free access to food and water.

### *In vivo* siRNA Transfection

The transfection of *NOX4* siRNA into rat brains *in vivo* was performed as previously described (Feng et al., 2015; Lu et al., 2019). Briefly, the rats were deeply anesthetized, and *NOX4* siRNA (sc-61887, Santa Cruz Biotechnology, United States) or control scramble siRNA (sc-37007, Santa Cruz Biotechnology, United States) premixed with transfection reagent (EntransterTM-*in vivo*, 18668-11-1, Engreen Biosystem co., LTD, Beijing, China) was injected into the lateral ventricle (1.5 mm posterior, 1.0 mm lateral, and 4.0 mm below the dural surface of the bregma). The microsyringe was left in place for 10 min after the intracerebroventricular injection and then slowly withdrawn. The Sham group received a burr hole, and an equivalent volume of saline was injected. The rats were kept under anesthesia throughout the procedure, as previously mentioned.

### Experimental Design

Experiments were conducted using a rat model of collagenase type IV-induced ICH. This study strictly followed the control principle, randomization principle, and blinding principle of animal experiments, and all animal groups were determined by drawing lots for complete randomization. The induction of the rat ICH model and sample acquisition were accomplished by one group of authors, while samples were detected and analyzed by another group of authors who were not involved in the induction of the rat ICH model and sample acquisition.

In the first batch, 90 rats (90/94 rats survived the surgery) were used to verify the expression of NOX4 after ICH. The rats were randomly and equally divided into 9 groups (*n* = 5 rats per group): the Sham group and 8 groups based on different timepoints after ICH (3, 6, 12 h, 1, 2, 3, 5, and 7 days). At a predetermined time, the rats were euthanised by overdose with narcosis (60 mg/kg; stock: 0.5 g sodium pentobarbital dissolved in 100 ml of sterile normal saline). Tissue from the haematoma on the haemorrhagic side of the rat brain was collected for analysis (real-time polymerase chain reaction (RT-PCR), western blot, immunohistochemistry).

In the second experiment, 15 rats (16 rats underwent the operation, 15 rats survived) were used to explore the localization of NOX4 expression in the central nervous system. The rats were euthanised at the peak NOX4 expression timepoint (3 days), and brain tissue was collected and sectioned for analysis (immunofluorescence double staining).

In the third experiment, 72 rats (76 rats underwent the surgery, and 72 survived) were used to investigate the relationship between NOX4 expression levels and oxidative stress after ICH. Rats were randomized into four groups (*n* = 18 rats per group): Sham group, ICH group, ICH + control scramble siRNA group, and ICH + NOX4 siRNA group. All rats in this experiment were euthanised at 3 days after ICH for immunohistochemistry and dihydroethidium (DHE) fluorescent probe detection (*n* = 3 rats per group), western blot analysis (*n* = 5 rats per group), enzyme-linked immunosorbent assay (*n* = 5 rats per group), and H_2_O_2_ content analysis (*n* = 5 rats per group).

In the fourth experiment, 48 rats (55 rats underwent the surgery, 48 rats survived) were used to study the relationship between NOX4 expression and neuronal apoptosis after ICH. Rats were randomly divided into 4 groups (*n* = 12 rats per group) and subjected to neurological behavior tests (*n* = 12 rats per group). As mentioned above, all rats in the experiment were sacrificed on day 3, the peak expression timepoint of NOX4, for immunofluorescence staining analyses and terminal deoxynucleotidyl transferase dUTP nick-end labeling (TUNEL) staining analyses (*n* = 5 rats per group), and western blot analyses (*n* = 5 rats per group).

In the fifth experiment, 72 rats (72/78 rats survived after the operation) were used to study the effects of *NOX4* siRNA interference on destruction of the blood-brain barrier following SBI. The rats were divided into four groups as described above. All rats were decapitated, and their brain tissues were harvested to perform western blotting (*n* = 5 rats per group), immunofluorescence staining (*n* = 5 rats per group), brain water content analyses (*n* = 5 rats per group), and Evans blue (EB) dye extravasation assessment (*n* = 3 rats per group).

### Antibodies

Anti-NOX4 antibody (ab133303), anti-Iba-1 antibody (ab15690), anti-8-Oxo-2′-deoxyguanosine (anti-8-OHdG) antibody (ab48508), anti-3-Nitrotyrosine (3-NT) antibody (ab61392), anti-Bcl-2 antibody (ab59348) were purchased from Abcam (Cambridge, MA, United States). Anti-NEUN antibody (#MAB377) and anti-glial fibrillary acidic protein (anti-GFAP) antibody (#MAB360) were purchased from Merck Millipore (Bedford, MA, United States). Anti-Bax antibody (#14796) and anti-caspase-3 antibody (#14220) were purchased from Cell Signaling Technology (Danvers, MA, United States). Anti-ZO-1 antibody (21773-1-AP), anti-occludin antibody (27260-1-AP), anti-matrix metalloproteinase-9 (anti-MMP-9) antibody (10375-2-AP), and anti-NEUN antibody (26975-1-AP) were purchased from Proteintech Biotechnology (Rosemont, IL, United States). Anti-claudin-5 antibody (#AB40754) was purchased from Absci Biotechnology (Vancouver, WA, United States). Anti-CD34 antibody (sc-74499) was purchased from Santa Cruz Biotechnology (Santa Cruz, CA, United States).

### Western Immunoblotting

Western blotting was performed as previously described (Liu L. et al., 2016). First, brain tissues were weighed and homogenized, mixed with RIPA lysis buffer (Shanghai Beyotime Biotechnology co., LTD, Shanghai, China), and placed on ice for 30 min. The lysates were centrifuged for 15 min at 12,000 *g* at 4°C and the supernatants were collected. Protein concentration was determined using a bicinchoninic acid (BCA) assay kit (Shanghai Beyotime Biotechnology co., LTD, Shanghai, China). Protein (50 μg) from each sample was separated using electrophoresis on 8–12% sodium dodecyl sulfate polyacrylamide gel electrophoresis (SDS-PAGE) gels at a constant voltage, and then transferred to polyvinylidene fluoride (PVDF) membranes using a wet electro transferring unit (Bio-Red, California, United States). The membranes were blocked with 5% bovine serum albumin (BSA) for 1 h at room temperature and then incubated with primary antibodies overnight at 4°C. This was followed by the incubation of the membranes with species-appropriate secondary antibodies at room temperature for 1 h. The PVDF strips were imaged using an ECL chemiluminescence system (Clinx Science Instruments Co., Ltd, Shanghai, China). The signal intensity of the primary antibody bands was quantitatively analyzed using the Image Lab software (Bio-Red, California, United States) and normalized to the intensity of the loading control, i.e., anti-Glyceraldehyde 3-phosphate dehydrogenase (anti-Glyceraldehyde 3-phosphate dehydrogenase (GAPDH) antibody (ab181602, Abcam, Cambridge, MA, United States).

### Immunofluorescence Staining

Immunofluorescence staining was performed as previously described (Paul et al., 2019). Specifically, formalin-fixed paraffin-embedded brain tissue was sectioned at a thickness of 4 μm. Following deparaffinisation and rehydration through xylene and a decreasing gradient series of alcohol solutions, the sections were blocked using 5% BSA at room temperature for 1 h, and incubated overnight with primary antibody (anti-rabbit NOX4 antibody, 1:200; anti-mouse GFAP antibody, 1:400; anti-mouse NEUN antibody, 1:250; anti-mouse CD34 antibody, 1:100; anti-mouse 8-OHdG antibody, 1:200; anti-mouse ZO-1 antibody, 1:200; anti-mouse Claudin-5 antibody, 1:200; anti-rabbit NEUN antibody, 1:200) at 4°C. Next, the samples were washed in phosphate buffered saline (PBS) buffer three times and then labeled with a fluorescence secondary antibody (Anti-mouse IgG (H+L), F(ab')2 Fragment (Alexa Fluor®555 Conjugate); Anti-rabbit IgG (H+L), F(ab')2 Fragment (Alexa Fluor®488 Conjugate); Anti-mouse IgG (H+L), F(ab')2 Fragment (Alexa Fluor®488 Conjugate); Anti-rabbit IgG (H+L), F(ab')2 Fragment (Alexa Fluor®555 Conjugate); Cell Signaling Technology, Danvers, MA, United States) at room temperature for 1 h, and counterstained using 4′6-diamidino-2-phenylindole (DAPI) for 10 min. Illumination and imaging were performed using a fluorescence microscope (Leica DMI8, Leica Microsystems Co., LTD, Germany). The number of positive cells in the haematoma region was counted in a blinded manner using the manual counting program included in the Image J software (National Institutes of Health, United States). Results are expressed as the percentage of positively stained cells. The fluorescence immunoreactive overlap of the samples was analyzed by Line scan analysis in Image J (National Institutes of Health, United States).

### Immunohistochemical Staining

Immunohistochemical staining was performed as previously reported (Shanbhag et al., 2019), with minor modification. Briefly, the coronal paraffin-embedded sections (4 μm thickness) of the rat brain were prepared in advance, dewaxed at 68°C for antigenic thermal repair, and incubated at room temperature with H_2_O_2_ to remove endogenous peroxidase. The sections were then incubated with 5% BSA at room temperature for 1 h to seal the non-specific epitope, and the samples were incubated overnight with primary antibodies (anti-rabbit NOX4 antibody, 1:400; anti-3-NT antibody, 1:200) at 4°C. Sections were washed with PBS buffer the following day, after which they were incubated first with biotinylated goat anti-rabbit IgG secondary antibodies for 25 min, and then with horseradish peroxidase (HRP)-streptavidin reagent for 25 min. Finally, 3,3-diaminobenzidine (DAB) was used to test the immunoreactivity of the sections and haematoxylin was used for counterstaining. The samples were examined with an open field microscope (Leica-DM2500, Leica Microsystems Co., LTD, Germany). The number of immune-positive cells in the haematoma region was counted in a blinded manner and the result was expressed as the percentage of positively stained cells, the counting of positively stained cells was completed using the manual counting program included in the Image J software (National Institutes of Health, United States). Overall, ten non-overlapping high-power visual fields around the haematoma were randomly imaged from three non-consecutive sections per animal, and the number of positively stained cells and total number of cells around the haematoma were counted under high-power objectives, the arithmetic mean of positively stained cells in ten high-power visual fields is used as data for statistical analysis. Immunopositive cells were counted by two blinded pathologists and graded according to their chromogenic intensities.

### RT-PCR

Total RNA was extracted from brain tissue using TRIZOL reagent (Invitrogen, Carlsbad, CA, United States). Quantitative RT-PCR was conducted with FastKing RT Kit and SuperReal PreMix Plus kit (SYBR Green) (KR116-02 and FP205-02, TIANGEN Biotech Co., LTD, Beijing, China), according to the manufacturer's instructions. The mRNA level of *GAPDH* was used as the internal control. The PCR program included 1 cycle of 95°C for 15 min, followed by 40 cycles of 95°C for 10 s, 60°C for 20 s, and 72°C for 30 s. Each sample was tested three times. The primer sequences used in this study are listed below.

*GAPDH*, Forward 5′-GATGCTGGTGCTGAGTATGRCG-3′ and Reverse 5′-GTGGTGCAGGATGCATTGCTCTGA-3′.

*NOX4*, Forward 5′-CAGAGACAAAAAGGGAGTAACTATT-3′ and Reverse 5′-TCACCTTTAGCTGGCAGACC-3′.

### TUNEL Staining

Terminal deoxynucleotidyl transferase dUTP nick end labeling (TUNEL) staining of coronal sections of the brain was used to analyse apoptosis, according to the manufacturer's instructions (*in situ* Cell Death Detection Kit, Roche, Germany), as previously described (Lin et al., 2019). First, the brain sections were first dewaxed (at 68°C for 1.5 h) and then treated with a gradient series of alcohol solutions to restore tissue moisture. The sections were then mixed with the TUNEL reactant and incubated at 37°C for 1 h. After washing in PBS three times, the samples were covered with fluorescence decay-resistant medium containing DAPI and covered with a cover slide. A fluorescence microscope (Leica DMI8, Leica Microsystems Co., LTD, Germany) was used to observe the TUNEL-positive cells in each section; an observer blinded to the experimental conditions analyzed the results.

### DHE Staining

The dihydroethidium (DHE) staining method for frozen sections was performed as previously described (Abe et al., 2018). The frozen coronal sections were rewarmed and incubated with dihydroethidium diluted in PBS (Sigma, Santa Clara, CA, United States) at room temperature for 30 min. The dye was then removed, and nuclei were counterstained with DAPI. Visualization and image capturing were performed using a Leica confocal microscope (Leica-SP8, Leica Microsystems Co., LTD, Germany).

### ELISA

Rats were deeply anesthetized and decapitated on day 3 after surgery. The tissue around the intracerebral haematoma was dissected. After homogenisation, the supernatant was aspirated and stored at −80°C. According to the manufacturer's instructions, the levels of superoxide dismutase (SOD), malondialdehyde (MDA), and glutathione peroxidase (GPx) were determined by double antibody sandwich ELISA array. Next, the prepared tissue supernatant was added to the monoclonal antibody enzyme, and the biotin-labeled SOD/MDA/GPx antibodies were combined with streptavidin-HRP to form an immune complex, which was then incubated at 37°C in a dark environment for 1 h. After adding the chromogenic substrate 3,3′,5,5′-tetramethylbenzidine (TMB), the ELISA plate was placed on the microplate reader and the absorbance of the samples was read at 450 nm; the optical density (OD) value of each well was recorded.

### Measurement of H_2_O_2_ Content

The content of H_2_O_2_ in brain tissue after ICH was determined using a H_2_O_2_ assay kit (Nanjing KeyGEN BioTECH Co., LTD, Nanjing, Jiangsu, China) according to the manufacturer's instructions.

### Neurological Scoring

On day 3 after ICH, the neurological functions of 12 rats in each group were tested to assess the extent of behavioral impairments. The scale score was assessed by two trained surveyors. As per previously published literature (Wang et al., 2012), appetite, activity, and neurological deficits of the rats were assessed (Table 1).

**Table 1:** Scoring system for neurobehavioral testing.

	**Behavior**	**Score**
Appetite	Finished meal	0
	Left meal unfinished	1
	Scarcely ate	2
Activity	Walk and reach at least 3 corners of the cage	0
	Walk with some stimulation	1
	Almost always lying down	2
Deficits	No deficits	0
	Unstable walk	1
	Unable to walk	2

### Measurement of Brain Water Content

Brain water content was measured based on dry and wet specific gravity as previously described (Yang et al., 2016). Briefly, on day 3 after ICH, each group of rats was deeply anesthetized and decapitated. The whole brain was removed and carefully divided into five parts, namely, the ipsilateral cerebral cortex and basal ganglia, contralateral cerebral cortex and basal ganglia, and cerebellum, which was used as an internal control. Each part was placed on pre-prepared weighed homogenized aluminum foil and weighed using an electronic analysis balance, and then, the tissues were dried at 100°C for 24 h in an oven to obtain the dry weight. Brain water content (%) was calculated as [(wet weight–dry weight)/wet weight] × 100%.

### Evaluation of Blood-Brain Barrier Permeability

Determination of blood-brain barrier permeability was evaluated via Evans blue dye (Sigma, Santa Clara, CA, United States) extravasation as previously described with minor modifications (Zeng et al., 2017). Briefly, rats were anesthetized and then, a 2% EB solution in normal saline (4 ml/kg) was administrated intravenously through the femoral vein. After allowing it to circulate for 2 h, an intracardiac perfusion with 250 ml of 0.9% normal saline was performed under deep anesthesia to clear the EB dye from the cerebral circulation. Subsequently, the rat brains were removed and photographed.

### Statistical Analysis

Statistical analyses were performed using SPSS (version 20; IBM Corp, Armonk, NY, United States), and GraphPad Prism 8 software (GraphPad, San Diego, CA, United States) was used to generate graphs. All data were tested for normality using Shapiro–Wilk test to make sure they were normally distributed prior to analysis of variance (ANOVA). No tests were performed for outliers and no data were excluded from the analysis. Parametric data were analyzed using one-way ANOVA followed by the Tukey's *post hoc* test and expressed as the mean ± standard error of the mean (SEM). Non-parametric data were analyzed by Kruskal–Wallis test, along with Dunn's *post hoc* tests, and are presented as the median and the interquartile range (25th percentile, 75th percentile). Neurobehavioural scoring results are presented as the median with the interquartile range. Differences with *p* < 0.05 were considered statistically significant. Sample sizes (*n* = 5) for animal studies were determined by power calculations in R language based on pilot studies or the literature (α = 0.05, power > 0.75).

## Results

### NOX4 Expression Is Increased in the Injured Basal Ganglia Following ICH

**Figure 1:**
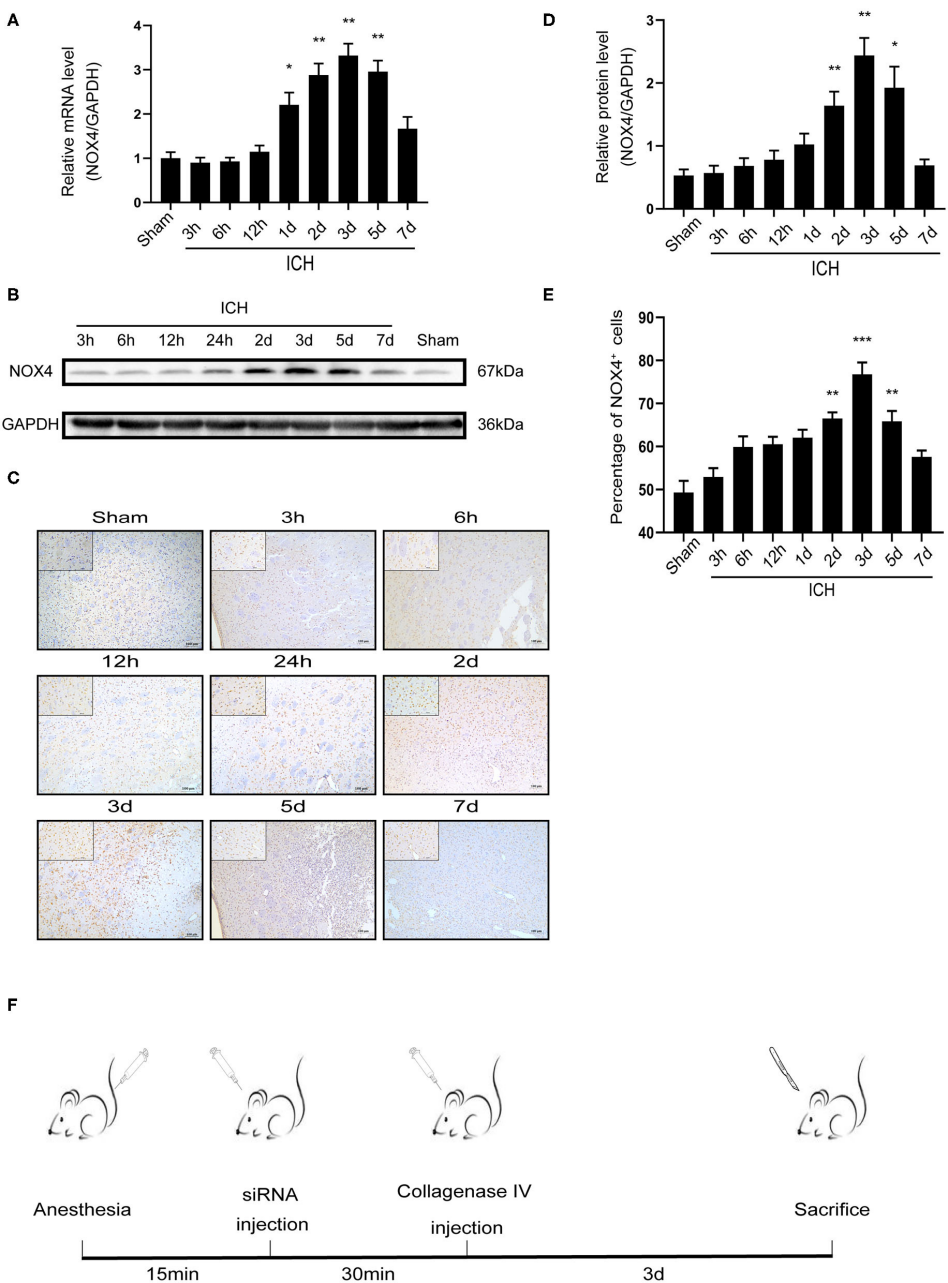
ICH increased the expression of NOX4 around the haematoma region in the basal ganglia. **(A)** *NOX4* mRNA expression levels at different timepoints after ICH. Relative mRNA levels (*NOX4/GAPDH*) were quantified. Values are expressed as mean ± SEM. *p < 0.05, **p < 0.01 Sham vs. ICH timepoint. *n* = 5 rats/group. **(B)** Representative western blot images showing the temporal pattern of NOX4 expression after ICH in the perilesional basal ganglia at 3, 6, 12 h, 1, 2, 3, 5, and 7 days following injury. GAPDH blot used for densitometry. **(C)** Representative immunohistochemical staining images showing the time-related protein expression trends of NOX4 in the perilesional basal ganglia at 3, 6, 12 h, 1, 2, 3, 5, and 7 days. Scale bar = 100 μm. **(D)** Quantified relative protein levels (NOX4/GAPDH). Values are expressed as mean ± SEM. *p < 0.05, **p < 0.01 Sham vs. ICH timepoint. *n* = 5 rats/group. **(E)** Quantification of NOX4+ cells in immunohistochemical staining images (represented as % of all haematoxylin+ cells). **p < 0.01, ***p < 0.001 Sham vs. ICH timepoint. *n* = 5 rats/group. **(F)** Anesthesia in rats, establishment of intracerebral hemorrhage model, *in vivo* siRNA transfection and rat sacrifice procedures.

After successfully inducing ICH in rats, RT-PCR was used to detect changes in *NOX4* mRNA expression at different timepoints, the samples required for RT-PCR, western-blot and immunohistochemical analysis were obtained from the injured area of ICH. As the results showed, the level of *NOX4* mRNA began to increase significantly at around 24 h, peaked at 3 days, and declined afterwards (Figure 1A). Meanwhile, NOX4 protein levels were measured in the tissues surrounding the haematoma at different timepoints following ICH. The results showed that the expression level of NOX4 began to rise slowly from 6 h after ICH, followed by a rapid increase to day 2, and reached its peak at day 3. Across the longer time spans (5–7 days), a gradual decrease was observed in the expression level of NOX4, and a return to baseline levels occurred at 7 days (Figures 1B,D). Immunohistochemistry confirmed the expression of the NOX4 protein after ICH. These findings were consistent with the western blotting results. The expression of the NOX4 protein increased sharply from 2 days after ICH, peaked at 3 days, and then slowly declined (Figures 1C,E). The results also indicated that the upregulation of *NOX4* mRNA occurred earlier than that of the NOX4 protein.

NOX4 is mainly expressed in neurons, astrocytes, vascular endothelial cells and microglia following ICH

**Figure 2:**
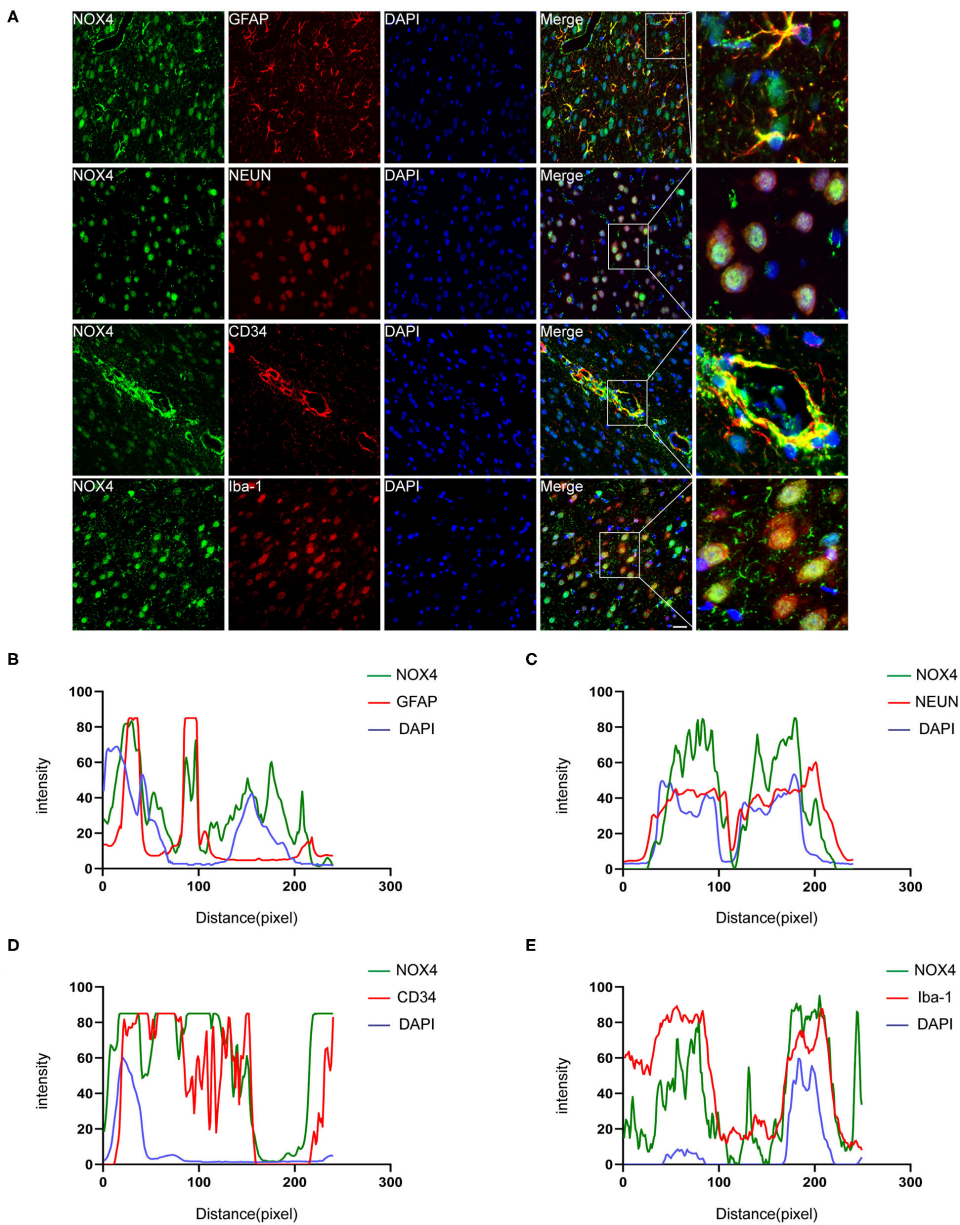
Immunofluorescence staining of NOX4 around the haematoma firmly colocalised in astrocytes, neurons, microglia and vascular endothelial cells. **(A)** Representative immunofluorescent images illustrated immunoreactivity of the injured basal ganglia region to NOX4 (green), GFAP (red), NeuN (red), Iba-1 (red), and CD34 (red). Nuclei are stained with DAPI (blue); the brain tissues were collected and used for immunofluorescence analysis on the third day after the rat ICH model was successfully induced. **(B–E)** Line-scan analysis on a representative NOX4+ cell in each group showing a strong overlap of the NOX4 intensity peak with GFAP, NeuN, Iba-1, and CD34, reflecting strong co-localization. Scale bar = 50 μm; *n* = 5 rats/group.

Immunohistochemistry and confocal microscopy imaging demonstrated that the elevated immunoreactivity of NOX4 was predominantly located in the neurons, astrocytes, vascular endothelial cells, and microglia in the injured basal ganglia. This observation is supported by a line-scan analysis of the channel intensity of NOX4/NEUN, NOX4/GFAP, NOX4/CD34, NOX4/Iba-1 and DAPI, which showed strongly overlapping NOX4 (green) and NEUN/GFAP/CD34/Iba-1 (red) intensity peaks (Figures 2A–E).

### The Overexpression of NOX4 Aggravates the Brain Damage in Rats With ICH

**Figure 3:**
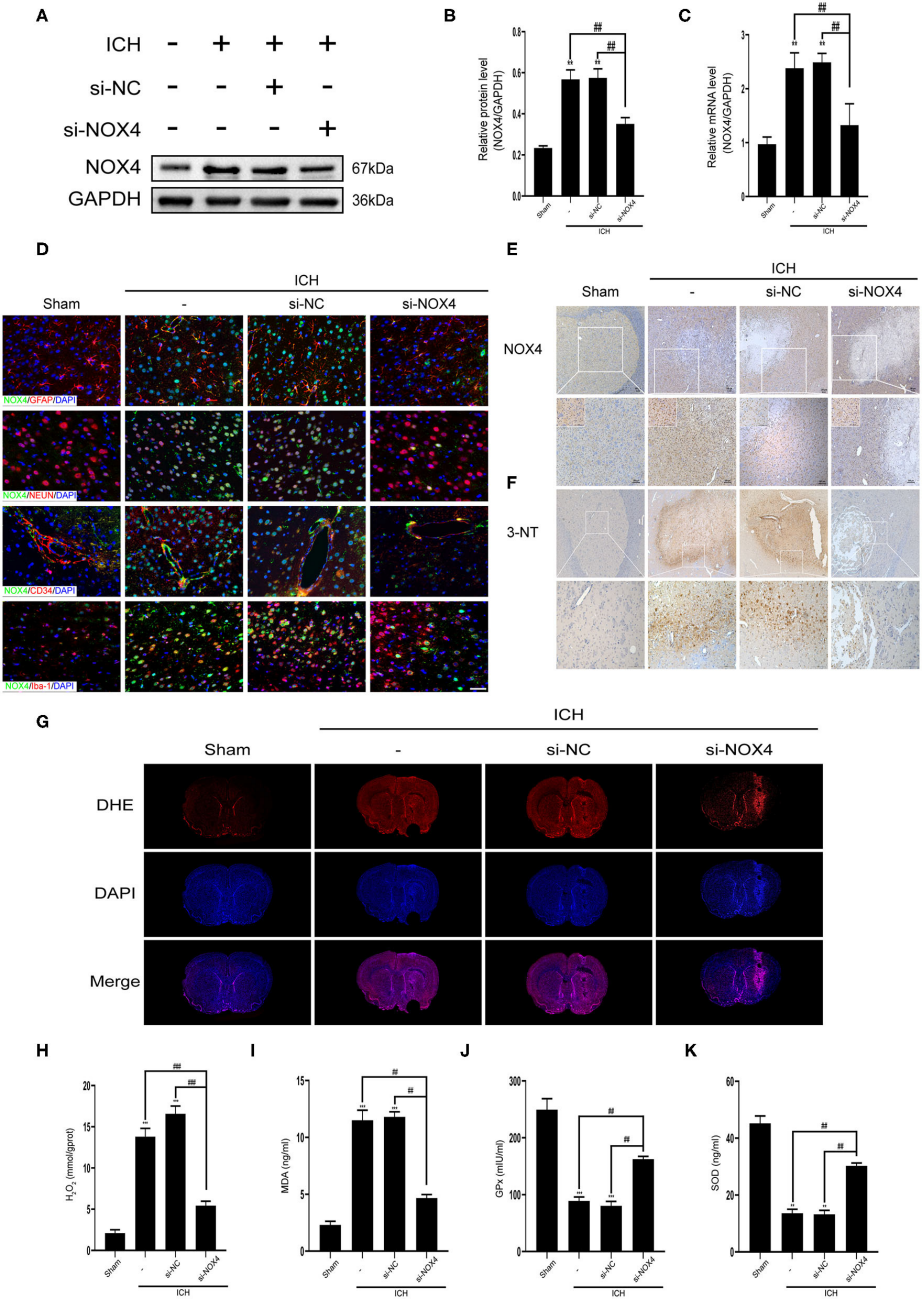
Inhibition of NOX4 expression led to a remarkable decline in the levels of oxidative stress in the central nervous system after cerebral hemorrhage. **(A)** Representative western blot images demonstrate NOX4 expression changes in tissues surrounding the haematoma before and after NOX4 siRNA treatment. GAPDH blot used for densitometry. **(B)** Quantitative western blot analysis showing a significant decrease in NOX4 expression around the haematoma after NOX4 siRNA treatment. NOX4 band densities were normalized to the densities of GAPDH. Values are expressed as mean ± SEM. **p < 0.01 vs. Sham. ^##^*p* < 0.01 si-NC vs. si-NOX4, ICH vs. si-NOX4. *n* = 5 rats/group. **(C)** NOX4 mRNA expression level before and after *NOX4* siRNA administration. Relative mRNA levels (NOX4/GAPDH) were quantified. Values are expressed as mean ± SEM. **p < 0.01 vs. Sham; ^##^*p* < 0.01 si-NC vs. si-NOX4, ICH vs. si-NOX4. *n* = 5 rats/group. **(D)** Representative immunofluorescence co-localization images showing that NOX4 (green) was knocked down in neurons (NEUN, red), astrocytes (GFAP, red), vascular endothelial cells (CD34, red), and microglial (Iba-1, red), Scale bar = 50 μm, *n* = 5 rats/group. **(E)** Representative immunohistochemical images illustrate changes in NOX4 protein expression before and after NOX4 siRNA treatment. Scale bar = 100 μm. **(F)** Representative immunohistochemical images showing the intensity of 3-nitrotyrosine expression around the haemorrhagic foci. Scale bar = 100 μm. **(G)** ROS in brain sections was examined by dihydroethidium (DHE) staining using a confocal microscope. **(H–K)** The levels of H_2_O_2_ **(H)**, MDA **(I)**, GPx **(J)**, and SOD **(K)** in brain homogenate were measured, respectively. Values are expressed as mean ± SEM. **p < 0.01, ***p < 0.001 vs. Sham; ^##^*p* < 0.01, ^###^*p* < 0.001 si-NC vs. si-NOX4, ICH vs. si-NOX4. *n* = 5 rats/group.

**Figure 4:**
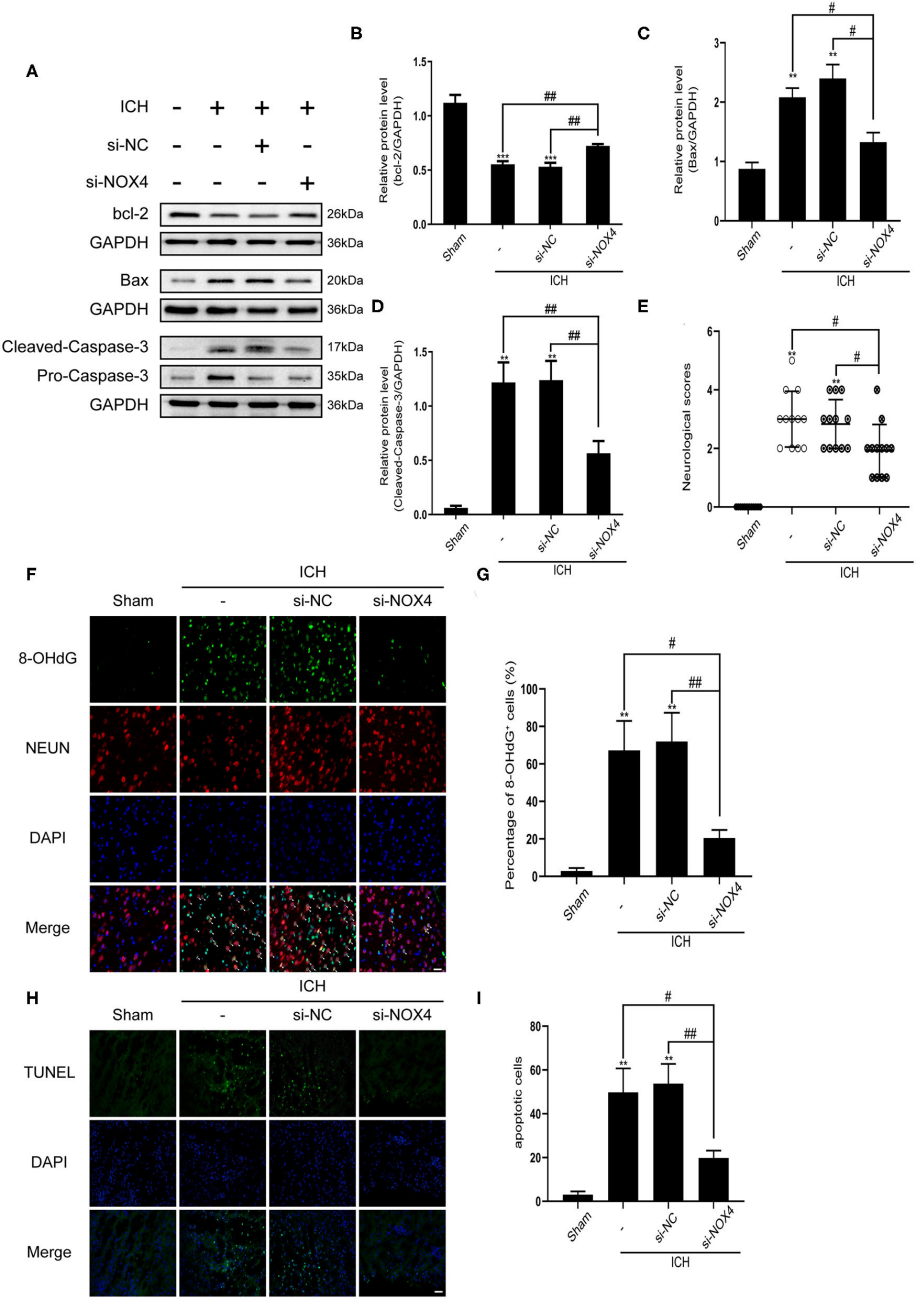
The level of apoptosis after cerebral hemorrhage was effectively reduced from inhibition of NOX4 expression. **(A)** Representative western blot images showing the effects of NOX4 siRNA on the expression of bcl-2, Bax, and caspase-3 after ICH. GAPDH blot used for densitometry. **(B–D)** Quantitative western blot analysis examined changes in the expression of bcl-2 **(B)**, Bax **(C)**, and caspase-3 **(D)** after silencing NOX4 by siRNA. Values are expressed as mean ± SEM. **p < 0.01, ***p < 0.001 vs. Sham. ^#^*p* < 0.05, ^##^*p* < 0.01 si-NC vs. si-NOX4, ICH vs. si-NOX4. *n* = 5 rats/group. **(E)** Neurological scores were evaluated as indicated in Table 1. **p < 0.01 vs. Sham; ^#^*p* < 0.05 si-NC vs. si-NOX4, ICH vs. si-NOX4. *n* = 12 rats/group. **(F,G)** Representative immunofluorescence images showing the immunoreactivity of the injured basal ganglia to NEUN (red) and 8-OHdG (green); Nuclei stained with DAPI (blue). Scale bar = 50 μm. Quantification of 8-OHdG+ cells (represented as % of all DAPI+ cells) in each group. **p < 0.01, vs. Sham. ^#^*p* < 0.05, ^##^*p* < 0.01 si-NC vs. si-NOX4, ICH vs. si-NOX4. *n* = 5 rats/group. **(H)** Representative TUNEL (green) staining of the perilesional basal ganglia of Sham, ICH, si-NC, and si-NOX4 treated rats. Nuclei stained with DAPI (blue). Scale bar = 100 μm. **(I)** The number of apoptotic cells was quantified, and data are expressed as the mean ± SEM, **p < 0.01 vs. Sham; ^#^*p* < 0.05, ^##^*p* < 0.01 si-NC vs. si-NOX4, ICH vs. si-NOX4. *n* = 5 rats/group.

**Figure 5:**
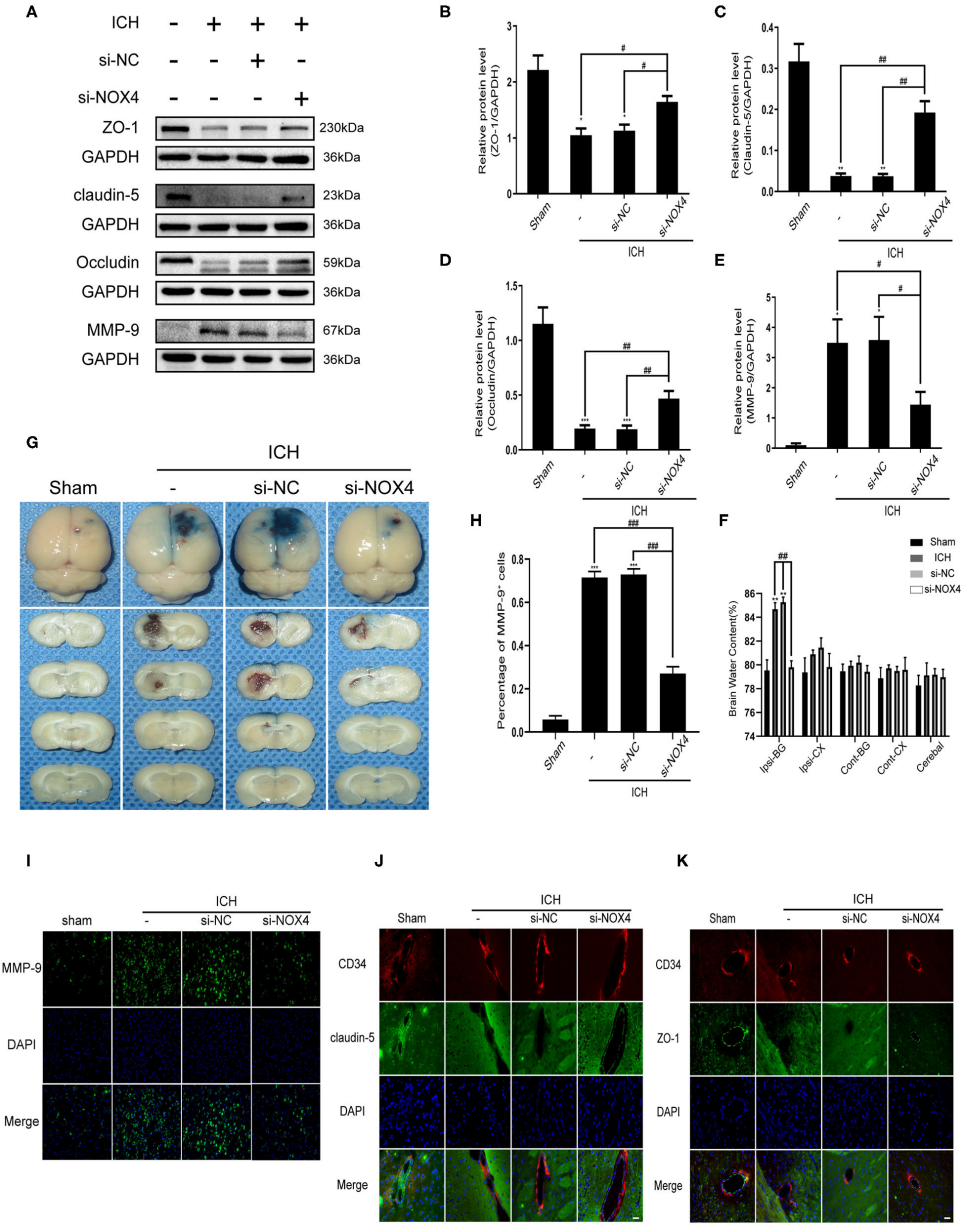
The destruction of blood-brain barrier function after ICH was improved by silencing the expression of NOX4. **(A)** Representative western blot images showing the effects of NOX4 siRNA on the expression of ZO-1, claudin-5, occludin, and MMP-9 after ICH. GAPDH blot used for densitometry. **(B–E)** Inhibition of NOX4 expression by a specific siRNA influenced the expression of ZO-1 **(B)**, claudin-5 **(C)**, occludin **(D)**, and MMP-9 **(E)** after cerebral hemorrhage. The relative protein levels were quantified with GAPDH as the loading control. Values are expressed as mean ± SEM. *p < 0.05, **p < 0.01, ***p < 0.001 vs. Sham. ^#^*p* < 0.05, ^##^*p* < 0.01 si-NC vs. si-NOX4, ICH vs. si-NOX4. *n* = 5 rats/group. **(F)** Brain water content. **p < 0.01 vs. Sham. ^##^*p* < 0.01 si-NC vs. si-NOX4, ICH vs. si-NOX4. *n* = 5 rats/group. **(G)** Representative pictures showing the extravasation of EB dyes. **(H,I)** Representative immunofluorescence staining showing MMP-9 (green), and nuclei labeled with DAPI (blue). Scale bar = 100 μm. The percentage of MMP-9+ cells was quantified, ***p < 0.001 vs. Sham; ^###^*p* < 0.001 si-NC vs. si-NOX4, ICH vs. si-NOX4. *n* = 5 rats/group. **(J,K)** Representative immunofluorescence staining showing ZO-1/Claudin-5 (green), CD34 (red), and nuclei labeled with DAPI (blue). Scale bar = 50 μm.

To clarify the role of NOX4 expression changes in central nervous system dysfunction caused by ICH, siRNA was used to block NOX4 expression at the peak timepoint of NOX4 expression post-ICH, i.e., 3 days (Figure 1F). Western blotting, immunohistochemistry, and RT-PCR were conducted to verify the feasibility of injecting *NOX4* siRNA into the lateral ventricle to eliminate the expression of NOX4, and the results showed that the blocking efficiency was ~40% (Figures 3A–E). In astrocytes, neurons, and vascular endothelial cells, the knockdown of NOX4 is significant, while the interference effect of NOX4 siRNA on the expression of NOX4 in microglia was more faintish than that in the above cells (Figure 3D). Compared with the rats from the Sham group, the rats with ICH showed significantly impaired neurobehavioural performance, which was dramatically improved by *NOX4* siRNA transfection *in vivo*. In contrast, the si-NC (injection of scrambled siRNA) group showed no obvious relief (Figure 4E). The impairment of blood-brain barrier function after ICH can lead to the aggravation of persistent cerebral oedema (Keep et al., 2008; Urday et al., 2015; Mittal and LacKamp, 2016); thus, we measured the cerebral water content and found that NOX4 knockdown substantially reduced the cerebral water content in rats and alleviated the degree of oedema (Figure 5F). In addition, the reduced amount of EB dye detected in the ipsilateral hemisphere indicated that the *NOX4* siRNA treatment significantly alleviated the extravasation of EB dyes (Figure 5G).

### Inhibition of NOX4 Expression Reduces ROS Production and Peroxidative Stress in Damaged Brain Tissues Following ICH

Previous studies have indicated that NOX4 and its downstream products play a key role in oxidative stress *in vivo* (Ma et al., 2018a). Therefore, we focused on the level of oxidative stress in the brain tissues of rats after silencing NOX4 activity. DHE staining of coronal brain sections in rats showed that the ROS content in brain tissues increased markedly after ICH, but this was completely reversed by the inhibition of NOX4 expression (Figure 3G). Similarly, the lipid MDA (peroxidation marker) and H_2_O_2_ (downstream product of NOX4) contents decreased substantially after NOX4 expression was diminished. Moreover, the levels of endogenous antioxidant enzymes, such as GPx and SOD, were further enhanced after NOX4 knockdown (Figures 3H–K). The levels of 3-NT were measured as indicators of post-translational oxidative damage following injury. Compared with the Sham group, there was a large amount of 3-NT expression around the haemorrhagic foci in the basal ganglia after cerebral hemorrhage, supporting the idea that cerebral hemorrhage increased tyrosine nitration. However, 3-NT levels were dramatically reduced by the blocking of NOX4 (Figure 3F).

### *NOX4* siRNA Administration Attenuates Neuronal Apoptosis in the Injured Brain Tissue Surrounding the Haematoma by Alleviating Oxidative Damage in Neurons After ICH Induction

Apoptosis of neurons was detected by TUNEL staining, which revealed a noticeable increase in the apoptosis of neurons around the haematoma after ICH. In the siRNA group, neuronal apoptosis was reduced (Figures 4H,I). The expression of 8-OHdG (a DNA oxidation damage marker) in neurons around the haematoma was measured using immunofluorescence; the results showed that 8-OHdG expression increased considerably after ICH and was associated with neurons. Interestingly, knocking down NOX4 noticeably reduced 8-OHdG production (Figures 4F,G). To further investigate the effect of the NOX4 protein on apoptosis regulation, the apoptotic protein caspase-3 was analyzed. Western blot results showed that cleaved caspase-3 levels in the ICH group were significantly higher than those in the Sham group. In response to treatment with *NOX4* siRNA, activation of caspase-3 was dramatically decreased, tracing the expression changes of the NOX4 protein. Bax and bcl-2 protein levels were significantly increased and decreased in the ICH group, respectively, consistent with the results for caspase-3, while NOX4 knockdown reversed the expression trends of these proteins (Figures 4A–D).

### The Damage to the Blood-Brain Barrier Aggravated by Oxidative Damage to Tight Junctions Was Reduced by NOX4 Knockdown Following ICH

The function of the blood-brain barrier is mainly maintained by tight junction proteins (Berndt et al., 2019), and the expression of a variety of tight junction proteins was assessed before and after the alteration of the expression of the NOX4 protein. The expression changes of ZO-1, occludin, and claudin-5 before and after ICH, as well as an injection of *NOX4* siRNA, were examined using western blotting. The results showed that the expression of ZO-1, occludin, and claudin-5 was remarkably downregulated after ICH compared to that in the control. After NOX4 was knocked down, the expression level of the tight junction proteins began to recover, showing a trend contrasting to that shown by the levels of oxidative stress in the brain tissue (Figures 5A–D). On the other hand, MMP-9, a molecule not conducive to the structural integrity of the blood-brain barrier, was also demonstrated to be associated with NOX4 (Figures 5A–E,H,I). Immunofluorescence staining located the tight junction around the endothelium accurately, and the expression level of the abovementioned tight junction proteins were in accordance with the western blotting results (Figures 5J,K). After the expression of NOX4 was knocked down, the brain water content decreased significantly, and the amount of EB dye exuding through the disrupted blood-brain barrier significantly decreased, indicating that the function of the blood-brain barrier was significantly improved (Figures 5F,G).

## Discussion

ICH is a subtype of stroke with a high mortality rate and disability and has attracted increasing attention over recent years. While the primary brain injuries incurred from ICH, such as haematoma, space occupying effects, and vasospasms, can be destructive, studies have shown that the effects of SBI last longer and are often more severe (Barzó et al., 1996). Importantly, an imbalance of oxidative stress may play an important role in the pathological process involved in SBI after ICH. In fact, studies have shown that elevated levels of oxidative stress caused by excessive production of ROS exerts a key role in the pathological progression of ischemic stroke and chronic neurodegeneration (Kelly et al., 2008; Leszek et al., 2016). In particular, the NOX family of proteins have become a promising target in oxidative stress research. In this report, we found that the expression of NOX4 was significantly upregulated after ICH. We further explored where NOX4 was expressed in the brain after ICH and determined if the knockdown of NOX4 expression could improve the degree of brain injury post ICH. Overall, our results support the targeting of NOX4 for the treatment of SBI after ICH.

NOX4 is situated in the plasma membrane, where it delivers electrons through the plasma membrane to produce ROS (Nishimura et al., 2016). Although first discovered in the kidney, NOX4 is also present in the central nervous system, mainly expressed in neurons and the vascular endothelium (Takac et al., 2012; Muñoz et al., 2019). Previously, researchers focused on the manifestation of the NOX2 subtype in the pathological evolution of various neurological diseases (Haslund-Vinding et al., 2017). However, recent studies have found that the expression of NOX4 is increased after acute brain injury, including ischemic stroke and traumatic brain injury (Casas et al., 2017). We now extend this to include ICH, as to the best of our knowledge, we are the first group to show that the expression of NOX4 was abnormally elevated after ICH. Additionally, we determined the cell localization of NOX4 in brain tissues after ICH, and found that in addition to neurons and vascular endothelial cells, NOX4 was also highly expressed in astrocytes.

**Figure 6:**
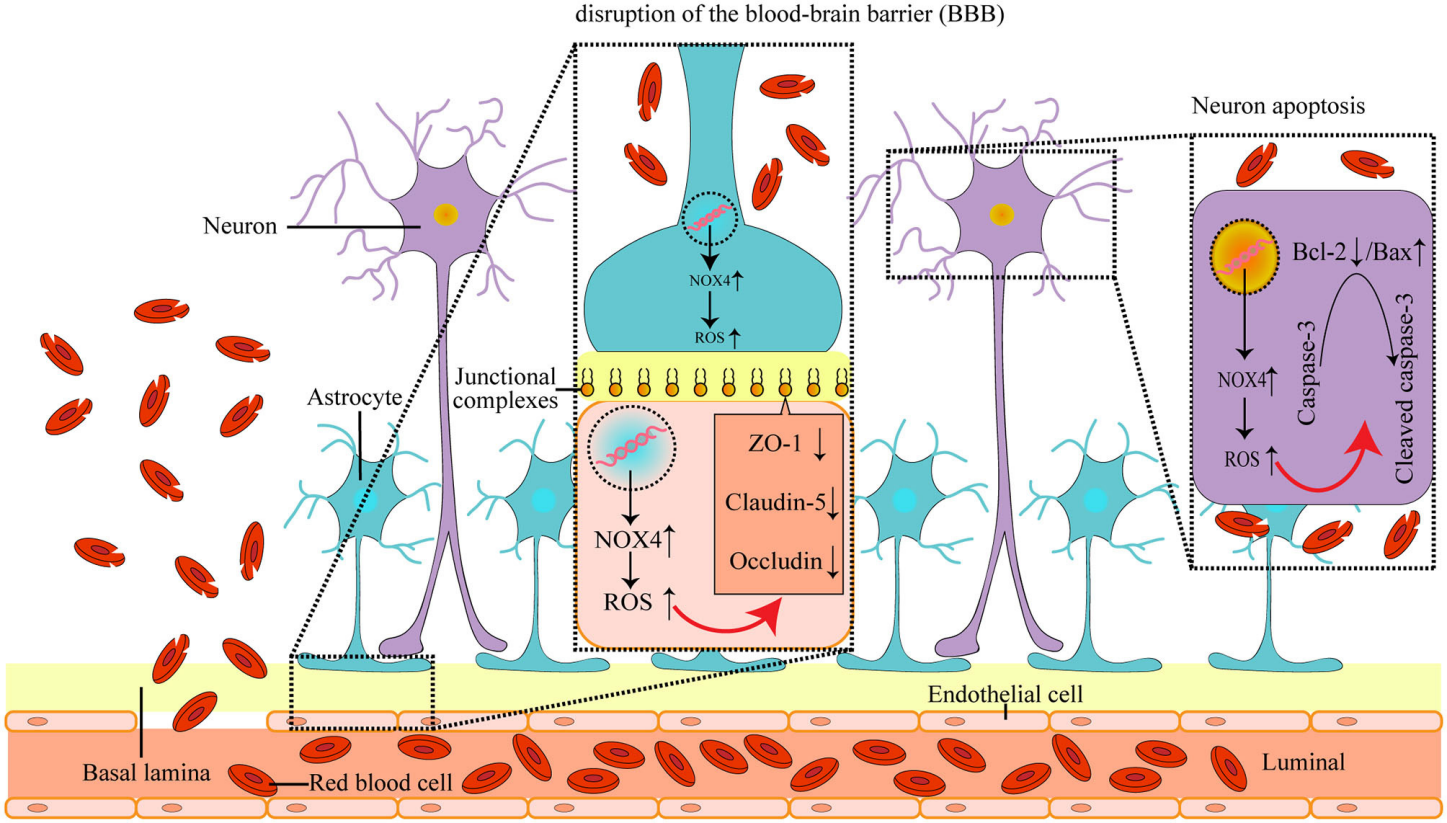
The mechanism of NOX4 in post-ICH SBI: the abnormal increase in NOX4 expression after ICH leads to the accumulation of ROS in neurons, astrocytes, vascular endothelial cells and microglia, and causes the peroxidation of lipids, proteins, and DNA, which further induces post-ICH neuronal apoptosis and neurological impairment, as well as loss of tight connections and damage of the blood-brain barrier in rats.

It has been reported that the expression of NOX4 in tissues is closely related to the corresponding level of oxidative stress (Zawada et al., 2015). Indeed, we found that the level of oxidative stress in the brain after ICH was reduced by the inhibition of NOX4, as evidenced by the significant reduction of lipid, protein, and DNA peroxidation. This suggests that NOX4 plays a critical role in the oxidative stress imbalance that occurs in the brain post-ICH (Figure 6).

Numerous studies have confirmed the existence of tissue apoptosis around a haematoma induced by ICH (Liu X. et al., 2016). Importantly, the imbalance of oxidative stress levels and the increased accumulation of ROS can directly impact the structure and function of biological macromolecules, such as lipids, proteins, and DNA, and thus, lead to cell apoptosis (Choi, 2018). Given that the elevated levels of oxidative stress observed after ICH were associated with the abnormally increased expression of NOX4, and that NOX4 is highly expressed in neurons, we speculated that NOX4 may mediate the apoptosis seen in neurons after ICH. In support of this, we found that knocking down NOX4 expression post-ICH decreased neuronal apoptosis and reduced cleaved caspase-3 levels, suggesting that NOX4 is involved in caspase-3-mediated cellular apoptosis occurring post-ICH. Previous research has also indicated that bcl-2, an anti-apoptotic molecule, can reduce the production of oxygen free radicals and lipid peroxides (Chien et al., 2001). Bax, an apoptotic counterpart to bcl-2, can antagonize bcl-2, and the relative ratio between Bax and bcl-2 is a key factor in determining the degree of inhibition of apoptosis (Li et al., 2018). We found that knocking down NOX4 expression after ICH increased the Bax levels and decreased the bcl-2 levels, suggesting that the abnormal expression of NOX4 after ICH leads to increased accumulation of intracellular ROS, and thus, affects the balance of bcl-2 and Bax expression, which leads to the activation of caspase-3 by splicing and apoptosis (Figure 6).

Apart from the neurological impairment caused by neuronal apoptosis, vasogenic brain oedema caused by the destruction of the blood-brain barrier after ICH is also a typical manifestation of SBI (Luh et al., 2010). In the process of neural development, the terminal foot of astrocytes covers a large number of cerebral capillaries, and the tight junctions between the endothelial cells of cerebral capillaries and astrocytes is the structural basis of the blood-brain barrier (Berndt et al., 2019). It has been reported that the induction of NOX4 is closely related to the destruction of the blood-brain barrier in ischemic stroke (Casas et al., 2017). Given our finding that NOX4 is expressed in vascular endothelial cells and astrocytes, we speculated that abnormal expression of NOX4 post-ICH may play a significant role in the destruction of the blood-brain barrier. A vast amount of literature indicates that the structural integrity and function of the blood-brain barrier are mainly dependent on tight junction proteins (Beauchesne et al., 2009; Haseloff et al., 2015). Therefore, we assessed the effects of the blocking of NOX4 post-ICH on the expression of tight junction proteins. We found a significant increase in the expression of ZO-1, claudin-5, and occludin, and effectively reduced brain water contents in rats, suggesting that abnormal intracellular oxidative stress caused by NOX4 post-ICH regulates the functional structural defects of the blood-brain barrier (Figure 6). Furthermore, it has been reported that ROS accumulation in brain tissues can activate MMPs and lead to the destruction of the blood-brain barrier (Mo et al., 2016). Similar to previous reports, we observed a remarkable decrease in MMP-9 expression after blocking NOX4. These results imply that NOX4 is important in the destruction of the blood-brain barrier as it enhances the level of oxidative stress in the brain and activates the expression of MMP-9. However, the mechanism whereby NOX4 regulates MMP-9 expression requires further exploration.

In conclusion, to the best of our knowledge, this study was the first to prove that the imbalance of oxidative stress mediated by NOX4 may be an important factor in the sustained development of SBI following ICH, and elucidate the possible molecular mechanism underlying the role of NOX4 in SBI via its cellular localization.

In order to successfully establish the ICH model but minimize the mortality of animals, we used collagenase type IV to induce ICH, which can stimulate sustained SBI after ICH. However, since hemorrhage in this ICH model is of the diffusive oozing type, the haematoma takes longer to form and does not induce the seriously acute space occupying effect (Elger et al., 1994; Clark et al., 1998). Therefore, future studies using different ICH models will be necessary to confirm our findings. One rat ICH model that can better simulate the pathological injury process of clinical ICH is the induced hereditary hypertension rat model, but this model is associated with a lack of stability and homogeneity. Therefore, the development and improvement of genetically deficient rat strains will permit the formation of an ideal experimental ICH model in the future. In this study, it was observed that the expression of NOX4 after ICH reached its peak on the third day. Interestingly, we noticed that the expression of NOX4 on the fifth day after ICH decreased slightly, though it still showed a significant upregulation compared to the Sham group; This is consistent with our results obtained from using immunofluorescence to evaluate the blocking efficiency of NOX4 in specific cells: *NOX4* siRNA cannot effectively block NOX4 expression in microglia, while the continuous migration and activation of microglia maintain the sustained expression of NOX4 in the hematoma region. Importantly, the expression of some inflammatory molecules can activate NOX protein family subunits, such as NF-κB and MAPKs (Morgan and Liu, 2011), and the persistent inflammatory reaction may be one of the reasons why the expression of NOX4 remains high on the fifth day. Furthermore, with the continuous infiltration and activation of microglia, the expression of NOX4 from these cells may also contribute to the persistently high expression state of NOX4.

Sadly, none of the ROS scavengers or ROS reducers that have been shown to be promising in the treatment of stroke in previous animal studies have been successfully translated to clinical settings (Bao et al., 2018). This is possibly due to the fact that anti-ROS therapy does not eliminate ROS production at the source. After ICH, ROS production is uncontrolled, leading to the continuous development of pathological processes. On the other hand, due to the action of the blood-brain barrier, anti-ROS molecules cannot quickly and effectively penetrate brain tissues. Even if a large dose of anti-ROS therapy is administered, it is still difficult to reach an effective therapeutic concentration in the brain. Therefore, fundamentally eliminating the excessive production of ROS in brain tissues after ICH is an urgent matter that needs to be addressed with regard to alleviating SBI and improving ICH treatment.

In this study, we demonstrated that NOX4 is an important factor mediating the pathophysiological alterations after ICH. We showed that knockdown of NOX4 expression after ICH can significantly reduce the level of oxidative stress and peroxidation damage, apoptosis, neurodegeneration, blood-brain barrier defects, and oedema in the brain. Given the failures of previous clinical trials, an effective clinical treatment is urgently needed to protect neurological function after ICH. Antioxidant stress therapy may represent a new treatment for ICH. NOX4 is the central node of oxidative stress regulation in the central nervous system and is expected to become a new therapeutic target for ICH. Importantly, the inhibition of NOX4 expression reduces the level of oxidative stress, while at the same time, maintains beneficial inflammatory responses (Matsushima et al., 2014). This supports the safety of antioxidant treatment strategies employing the inhibition of NOX4. In conclusion, our results further implicate NOX4 as a therapeutic target for ICH, help clarify the selection of a key time period for NOX4-targeted therapeutic strategies after the onset of ICH, and support the continued development of NOX4-specific inhibitors.

## Data Availability Statement

The raw data supporting the conclusions of this article will be made available by the authors, without undue reservation.

## Ethics Statement

The animal study was reviewed and approved by ethics committee of Southern Medical University.

## Author Contributions

JX and EH were involved in the conception, design, and writing of the manuscript. JX, BD, and SZ performed multiple experiments and collected, analyzed, and interpreted the data. WJ, ZX, and DT conducted statistical analyses and revised the manuscript. YC provided technical support, obtained funding, and was involved in the conception and design of the study, and the revision of the manuscript. All authors have read and approved the final manuscript.

## Conflict of Interest

The authors declare that the research was conducted in the absence of any commercial or financial relationships that could be construed as a potential conflict of interest.
